# Spin orbit torques induced magnetization reversal through asymmetric domain wall propagation in Ta/CoFeB/MgO structures

**DOI:** 10.1038/s41598-018-19927-5

**Published:** 2018-01-22

**Authors:** Jiangwei Cao, Yifei Chen, Tianli Jin, Weiliang Gan, Ying Wang, Yuqiang Zheng, Hua Lv, Susana Cardoso, Dan Wei, Wen Siang Lew

**Affiliations:** 1 Key Laboratory for Magnetism and Magnetic Materials of the Ministry of Education, Lanzhou University, Lanzhou, 730000 People’s Republic of China; 2 School of Physical and Mathematical Sciences, Nanyang Technological University, 21 Nanyang Link, Singapore, 637371 Singapore; 3 INESC Microsistemas e Nanotecnologias (INESC MN), Lisbon, 1000–029 Portugal; 4 Instituto Superior Tecnico, Universidade de Lisboa, Av. Rovisco Pais, Lisbon, 1000 Portugal; 5 Key Laboratory for Advanced Materials, School of Materials Science and Engineering, Tsinghua University, Beijing, 100084 China

## Abstract

The magnetization reversal induced by spin orbit torques in the presence of Dzyaloshinskii-Moriya interaction (DMI) in perpendicularly magnetized Ta/CoFeB/MgO structures were investigated by using a combination of Anomalous Hall effect measurement and Kerr effect microscopy techniques. By analyzing the in-plane field dependent spin torque efficiency measurements, an effective field value for the DMI of ~300 Oe was obtained, which plays a key role to stabilize Néel walls in the film stack. Kerr imaging reveals that the current-induced reversal under small and medium in-plane field was mediated by domain nucleation at the edge of the Hall bar, followed by asymmetric domain wall (DW) propagation. However, as the in-plane field strength increases, an isotropic DW expansion was observed before reaching complete reversal. Micromagnetic simulations of the DW structure in the CoFeB layer suggest that the DW configuration under the combined effect of the DMI and the external field is responsible for the various DW propagation behaviors.

## Introduction

Recently, current-induced highly-efficient magnetization reversal^1–3^ and fast domain wall (DW) motion^4–6^ by utilizing spin orbit torques (SOT)^7^ have drawn much attention for their potential application in magnetic memory^8–10^ and logic devices^11^. In heavy metal (HM)/ferromagnetic (FM)/Insulators (I) heterostructures with broken inversion symmetry, an in-plane current may induce SOT with both damping-like and field-like terms, resulting from spin Hall (SHE)^3^ and Rashba^1^ effects. Although the field-like term was non-negligible in most of the HM/FM/I structures, both theoretical and experimental works have suggested that the SHE mechanism by itself was sufficient to explain the current-induced magnetization switching and DW motion in these structures^3–6,12,13^. In the SHE regime, the spin current originating from the spin dependent scattering in the HM layer penetrates through the HM/FM interface and exerts a torque on the FM layer, which may induce deterministic magnetization switching of the FM layer^3^. The application of SOT-induced magnetization switching in magnetic random access memory (MRAM) prevents damage to the insulating layer from the large writing current, which remains a significant challenge for spin-transfer torque MRAM^8,9^. However, in the HM/FM/I structures with perpendicular magnetic anisotropy (PMA), theoretical and experimental work have verified that an external in-plane magnetic field is required for deterministic switching to break the symmetry along the current direction^3,14^. Although this in-plane field may in principle be supplied by an integrated bias permanent magnet, it is undesirable from a practical point of view^15^. Therefore, researchers have opted to reduce or eliminate the required external in-plane magnetic field by engineering the film stacks^15–18^.

From another perspective, understanding the role of the in-plane magnetic field in SOT-induced magnetization switching is equally as important for the application of SOT effect. Initially, a macrospin model was used to explain the role of the in-plane magnetic field along the current direction, which is required to break the symmetry of current-induced damping-like field with respect to the “up” or “down” magnetization states^3^. However, for devices of micron dimensions, a macrospin description is clearly inadequate to provide an accurate quantitative understanding of the reversal process because of the presence of the spatially nonuniform reversal process^3^. The current-induced DW depinning model proposed by Lee *et al*.^14^ gave a better understanding of the magnetization reversal process and the role of the in-plane field in SOT-induced magnetization switching. They suggested that the function of the field was to orient the magnetic moments within the DW to align a significant component parallel to the current flow, thereby allowing the torque from the SHE to produce a perpendicular equivalent field that can expand a reversed domain in all lateral directions. However, that does not explain why experimentally the required in-plane field for current-induced deterministic switching is only approximately 10%-25% of the effective field caused by the Dzyaloshinskii-Moriya interaction (DMI). In addition, the subsequent magneto-optical Kerr effect (MOKE) study of current-induced switching in HM/FM/I structures confirmed the DW depinning process driven by SOT. Nevertheless, how the DMI affects the current-induced DW propagation is still unclear. Moreover, the results from experimental observations of DW propagation process in similar film stacks are inconsistent^19–23^. An understanding of nucleation and SOT-induced DW propagation in HM/FM/I structures with DMI therefore remains incomplete.

In our study, a systematic analysis of SOT-induced magnetization switching in Ta/CoFeB/MgO structures under various in-plane magnetic fields was performed. The current-induced DW propagation process under various in-plane magnetic fields was observed using MOKE microscopy. Finally, by micromagnetic simulations of the DW structure with the inclusion of DMI effects, we identified the origin of the current-induced asymmetric DW propagation and the role of the in-plane field in SOT-induced magnetization reversal.

## Results

**Figure 1:**
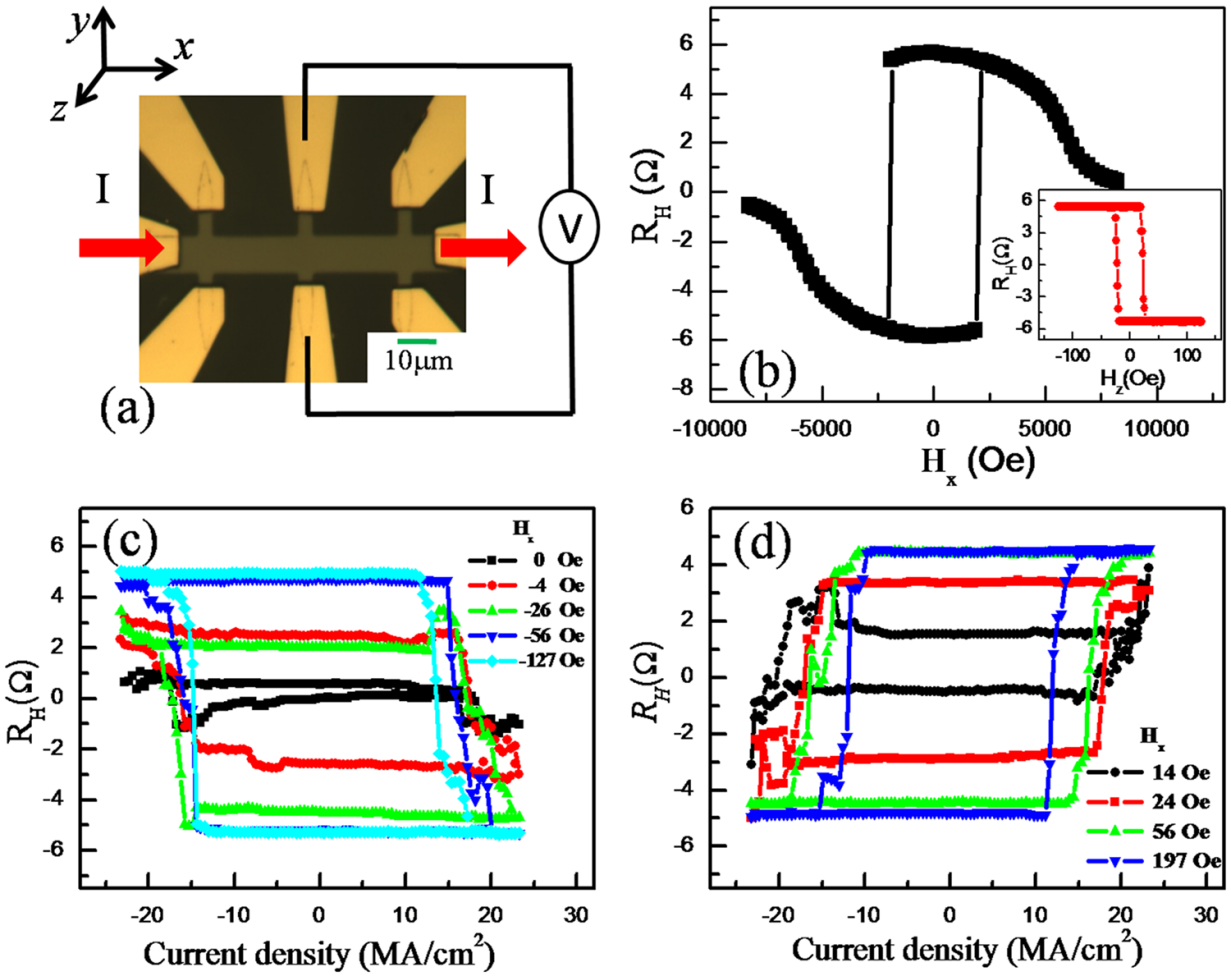
Anomalous Hall effect (AHE) and current-induced switching in the Ta/CoFeB/MgO structure. (**a**) Top-view photomicrograph of a device showing the configuration for AHE measurements, and coordinate system. (**b**) In-plane and out-of-plane (inset) magnetic field dependence of the Hall resistance *R*_*H*_. (**c**) and (**d**) show the measurement results for current-induced magnetization switching from *R*_*H*_ values for various constant in-plane magnetic fields *H*_*x*_. In measurements, a small current of 0.1 mA between two consecutive current pulses was used to detect the magnetization orientation in the devices.

A film stack with the structure of Ta (3 nm)/Co_20_Fe_60_B_20_ (1.3 nm)—hereafter denoted as CoFeB/MgO (1 nm)/Ta (1 nm) layers was deposited at room temperature on thermally oxidized Si substrates by using a magnetron sputtering system with a base pressure below 1.0 × 10^−7^ Torr. The film stack was subsequently patterned into eight-terminal Hall bar devices of differing dimensions by standard photolithography and ion milling techniques. A top view photomicrograph of a typical device is shown in Fig. 1(a). The current-induced magnetization switching was characterized from anomalous Hall effect (AHE) measurements and MOKE microscope images taken at room temperature. In the AHE measurement, the Hall resistance (*R*_*H*_), which is proportional to the perpendicular magnetization of CoFeB in the structures, was measured using a constant 100-μA bias current. A constant *R*_*H*_ value was subtracted from the original data to remove the offset of *R*_*H*_ resulting from the welding-spot misalignments of the voltage terminals. The square AHE loops shown in the inset of Fig. 1(b) indicate that the device has a strong PMA. The effective magnetic anisotropy field (*H*_*k*_^*eff*^) can be evaluated by fitting to the hard-axis magnetic field dependence of *R*_*H*_, which is around 7 kOe (Fig. 1(b)).

Next, we investigated the current-induced magnetization switching under different in-plane magnetic fields (*H*_*x*_) in a 10-μm-wide device. In this experiment, a series of current pulses with pulse width of 1 ms was applied to the devices to switch the magnetization. Between two consecutive pulses, a small current of 0.1 mA was used to determine the magnetization orientation. Figure 1(c,d) shows the current-induced magnetization switching under a constant *H*_*x*_ with different amplitude and direction. When an *H*_*x*_ of above 50 Oe is applied, the pulse current induces a deterministic magnetization switching, with positive current favoring *R*_*H*_ > 0. If *H*_*x*_ is reversed, the current-driven transitions are reversed, with positive current now favoring *R*_*H*_ < 0. Whereas only incomplete magnetization switching was observed with |*H*_*x*_| < 50 Oe and almost no switching occurs with |*H*_*x*_| < 10 Oe.

**Figure 2:**
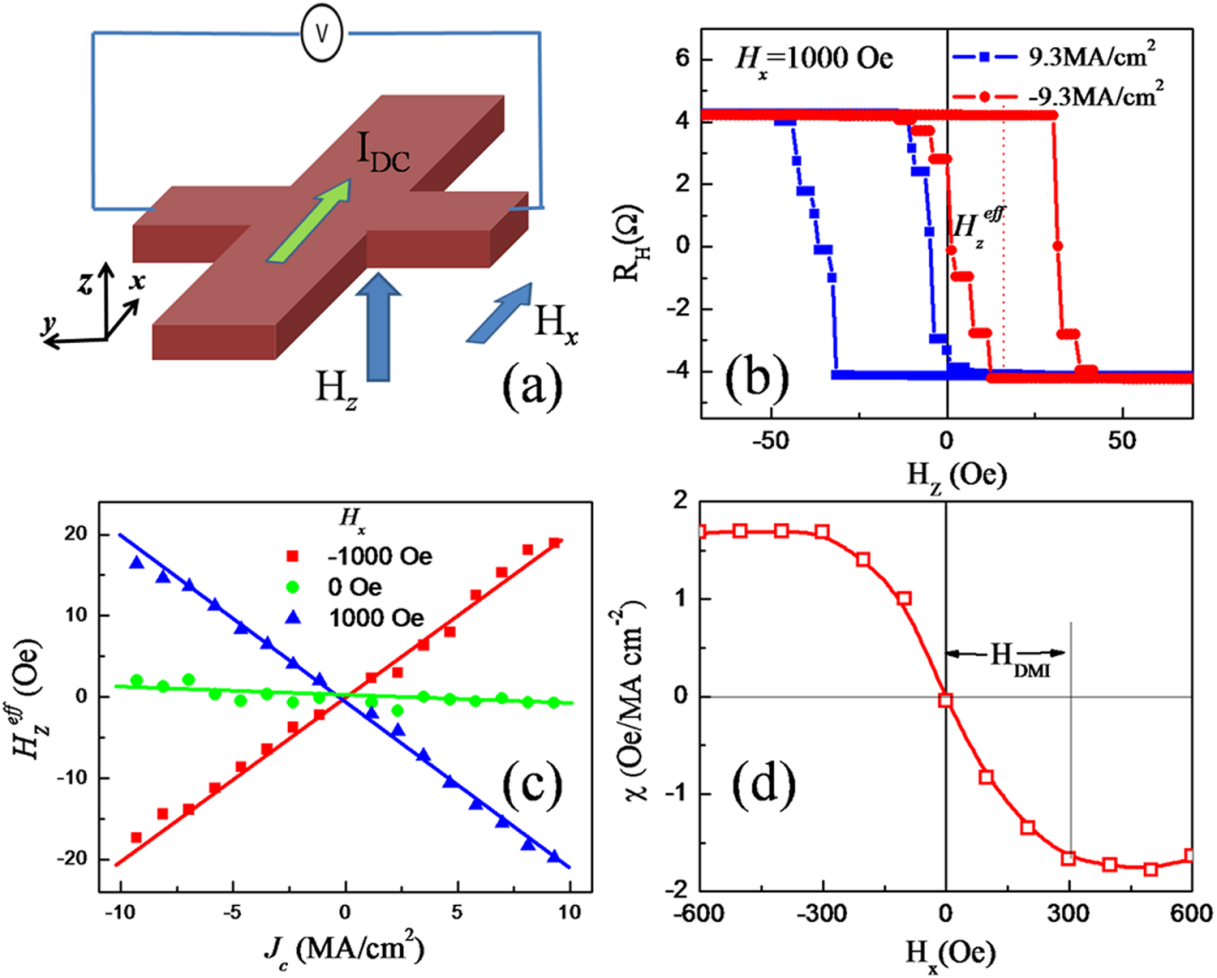
Measurements of spin orbit torque efficiency and DMI effective field. (**a**) Schematic of AHE measurements with an in-plane field *H*_*x*_ applied. (**b**) AHE loops with dc currents density *J*_c_ = ±9.3 MA/cm^2^ and an in-plane bias field *H*_*x*_ = 1000 Oe applied. (**c**) *H*_*z*_^eff^ as a function of *J*_c_ under different bias fields. (**d**) Measured effective *χ* as a function of applied in-plane field.

To understand the microscopic mechanism of SOT-induced magnetization switching under various *H*_*x*_, the function of the DMI at the HM/FM interface needs to be considered. Previous works have suggested that the chiral Néel domain walls (DWs) can be stabilized by the DMI in ultrathin films lacking inversion symmetry^5,6,24,25^. The current-induced magnetization switching and DW motion in HM/FM bilayers then can be explained by a SHE + DMI scenario^5,14^. Because the spin Hall angle of Ta is negative^2^, the SHE effective field produced by a negative current (−x direction) can be expressed as ${\overrightarrow{H}}_{SH}={H}_{SH}\hat{m}\times \hat{{\rm{y}}}$^14^. As a result, the vertical component of the equivalent field of the SHE is *H*_*z*_^*eff*^ = *H*_*SH*_*m*_*x*_. For chiral Néel DWs, the perpendicular component of the current-induced effective field (*H*_*z*_^*eff*^) at the DW can lead to DW motion but not domain expansion in the absence of *H*_*x*_ because of the opposite signs of *H*_*z*_^*eff*^ for up-down and down-up DWs. However, by applying a large enough *H*_*x*_ to overcome the effective DMI field (*H*_DMI_), the DW moments in the Néel-type walls are realigned parallel to *H*_*x*_. In this case *H*_*z*_^*eff*^ points along the same direction for both up-down and down-up walls and therefore facilitates both domain expansion and contraction, ultimately fulfilling the criteria for deterministic current-induced switching^14^. Conversely, in measuring the AHE with large current, current-induced *H*_*z*_^*eff*^ at the DW may compete with the applied perpendicular field (*H*_*z*_) and induce a considerable shift along the *H*_*z*_ axis in the *R*_*H*_ *vs H*_*z*_ loops^21^. Then the effective DMI field can be acquired by measuring the spin torque efficiency (i.e. *H*_*z*_^*eff*^ per current density) as a function of *H*_*x*_. As schematically shown in Fig. 2(a), we measured the *R*_*H*_ *vs H*_*z*_ loops in the Hall-bar devices as a function of applied dc current density (*J*_c_) and *H*_*x*_. *J*_*c*_ was obtained by the total current Representative *R*_*H*_ vs *H*_*z*_ loops with *H*_*x*_* = *1000 Oe and *J*_dc_ = ±9.3 MA/cm^2^ are shown in Fig. 2(b). The opposite loop shifts along the *H*_*z*_ axis of the hysteresis loops corresponding to opposite polarities of *J*_*c*_ indicate the presence of a current-induced *H*_*z*_^*eff*^ generated from the damping-like torque. From the current-induced *H*_*z*_^*eff*^ plotted against *J*_c_ with different amplitudes and polarities, as shown in Fig. 2(c), the linear variation provides a good estimate of *H*_*z*_^*eff*^ /*J*_*c*_. To verify that this measured *H*_*z*_^*eff*^ indeed stems from the SHE, we also measured the *H*_*z*_^*eff*^–*J*_*c*_ curves with *H*_*x*_ = −1000 and 0 Oe. By reversing the polarity of *H*_*x*_, the slope of *H*_*z*_^*eff*^ */J*_*c*_ is also reversed. This is consistent with the prediction from the SHE + DMI scenario^5,14^. In addition, a near-zero *H*_*z*_^*eff*^ */J*_c_ value at *H*_*x*_ = 0 Oe coincides also with the fact that no current-induced switching happening at zero *H*_*x*_ [Fig. 1(c)]. In Fig. 2(d), we summarized the measured effective field per current density (*χ* = *H*_*z*_^*eff*^*/J*_*c*_) as a function of *H*_*x*_. We find that *χ* increases quasi-linearly with *H*_*x*_ and saturates at *H*_*x*_ ≈ ±300 Oe, at which the DW moment in the Néel-type walls realign parallel to *H*_*x*_, and therefore the |*H*_*z*_^*eff*^*|* attains a maximum. Based on this model, we estimate *χ*_SHE_ ≈ 1.7 Oe*/*(MA*/*cm^2^) and |*H*_DMI_| ≈ 300 Oe for Ta (3)/CoFeB (1.3)/MgO(1) from the saturation value of *χ* and the saturation field, respectively. The low *χ*_SHE_ indicates a corresponding low spin Hall angle (0.06) of the Ta layer, but it is consistent with our results measured using harmonic voltage method^26^. The DMI constant (D) was estimated from H_DMI_ = D/(μ _0_M_S_Δ)^27^, where M_s_ is the saturation magnetization of the CoFeB layer (1200 emu/cc) and Δ the DW width obtained from ${\rm{\Delta }}=\sqrt{A/{K}_{eff}}$_,_ A = 16 pJ/m, *K*_*eff*_ = 4.2 × 10^5^ J/m^3^. The calculated DMI constant is around 0.22 mJ/m^2^, which is within the range of previously reported values in similar magnetic heterostructure systems^25,28,29^. According to the q-Φ model proposed by André Thiaville *et al*.^27^, the critical value of DMI to stabilize the chiral Néel DWs is given by $D{\rm{c}}=4{\rm{\Delta }}K/\pi$, where Δ is the DW width and K is the magnetostatic “shape” anisotropy that favors the Bloch wall, related to the “demagnetizing coefficient” N_x_ of the wall by $K={N}_{x}{\mu }_{0}{{M}_{s}}^{2}/2$. The calculated D_c_ is around 0.16 mJ/m^2^ for our Ta/CoFeB/MgO films, which confirm that the DMI in our films is high enough to stabilize the chiral Néel DWs.

**Figure 3:**
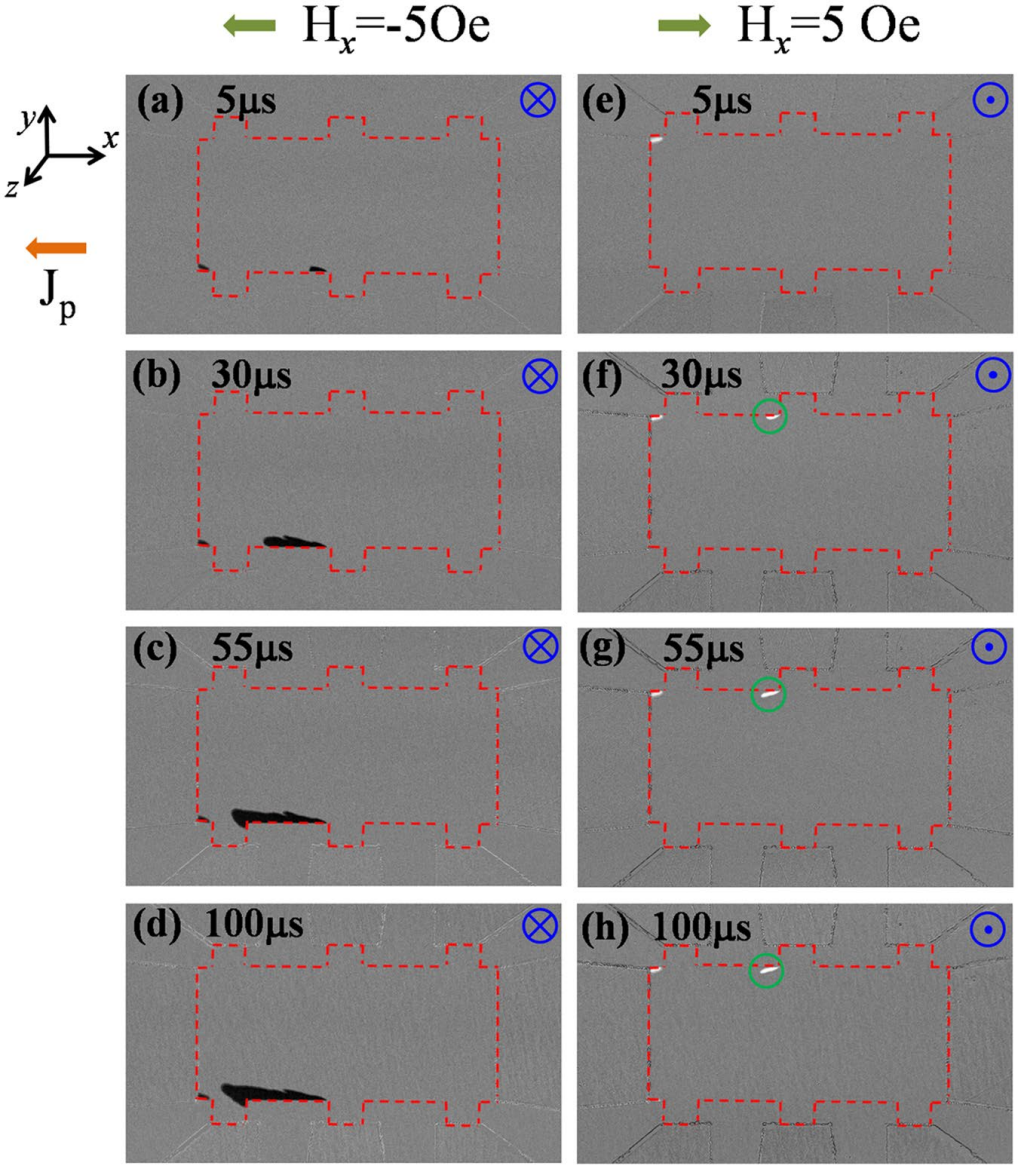
MOKE images of a Hall bar device after applying a series of current pulses (5 μs in duration and 10 MA/cm^2^ in current density for each pulse) in the presence of a small *H*_*x*_ of ±5 Oe. Before applying the current pulses, the device was pre-saturated with (left column) a downward magnetization (right column) upward magnetization. The direction of *H*_*z*_ for pre-saturating the sample and the applied total pulse duration before taking the images are given at the top right and top left of each panel, respectively. The green circles in the right column marked the area with tiny DW propagation.

To gain insights in the processes resulting in the current-induced magnetization reversal, we performed microscope imaging of the magneto-optical Kerr effect on a device with the same film stack structure but different dimensions (80 μm in width). In this experiment, the device was initially saturated by applying a perpendicular magnetic field (*H*_*z*_), either up or down; *H*_*z*_ was then removed and a constant *H*_*x*_ applied. A series of short current pulses (5 μs in duration for each pulse) was applied to the device to produce SOTs, thereby inducing nucleation with reversed magnetization and subsequent DW propagation in the magnetic layer. Immediately following each pulse, MOKE images were taken to monitor the magnetization status of the CoFeB layer. First, this procedure was performed with a pulse current density (*J*_*p*_) of 10 MA/cm^2^ at *H*_*x*_ = ±5 Oe. The corresponding MOKE images after applying the current pulses were shown in Fig. 3. In the images, the magnetic area of the device exposed under the microscope is outlined (red dotted lines) as a guide. Note that the reversed domains always nucleate along the bottom edge of the stripe after the first current pulse for the down-magnetized case (the left column in Fig. 3). If the initial out-of-the-plane magnetization state is reversed, nucleation is seen to occur on the top edge instead (Fig. 3(e,f)). In all observations, the Oersted field is always anti-parallel to the initial magnetization. This strongly suggests that, for small *H*_*x*_, the nucleation location is determined by the Oersted field generated by the in-plane charge current flowing along the device. From a numerical calculation of the Oersted field produced by the current^20^, the vertical component of the Oersted field is found to peak but with opposite polarity at the two long edges of the device. Its peak value at the edges is about 7.2 Oe with *J*_*p*_ = 10 MA/cm^2^ for 80-μm-wide devices. As the Oersted field is still lower than the measured nucleation field (i.e., coercivity) of the sample of about 15 Oe, we believe that the effective field produced by the SOT also contributes in nucleating the reversed domain. After applying several pulses, the left-hand side of the DW propagates slowly along the charge current direction for both down-magnetized (left column) and up-magnetized (right column) configurations. However, we did not observe transverse and rightward DW propagation after applying 20 pulses (100 μs duration in total) for both cases. The mechanism underlying the asymmetric DW propagation induced by the current is discussed in detail below after analyzing the DW structure and current-induced SHE effective field in the structure.

**Figure 4:**
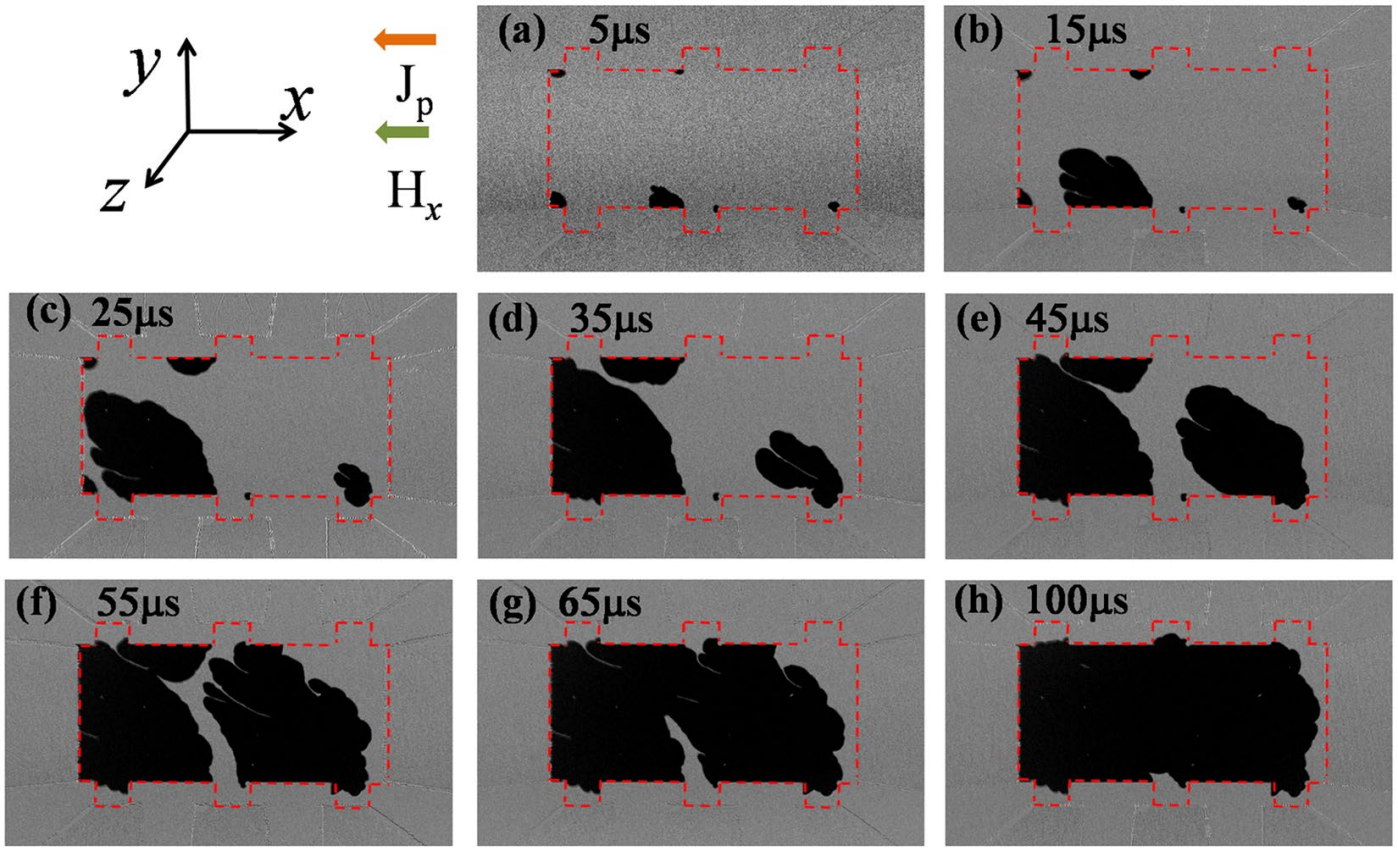
Same as Fig. 3 but in the presence of a medium *H*_*x*_ = −145 Oe. The device was pre-saturated with a downward magnetization and negative current flowing leftward.

When |*H*_*x*_| was increased to 145 Oe, a different current-induced domain nucleation and DW propagation process was observed. The domains are nucleated at both top and bottom edges of the stripe after the first current pulse, as shown in Fig. 4(a). This indicates that the SOT effective field alone is sufficient to nucleate domains in contrast to the case with H_x_ = ±5 Oe where the Oersted field played a significant role. In accordance with the theory of SHE, the vertical component of the equivalent spin Hall field is *H*_*SH*,*z*_ = *H*_*SH*_*m*_*x*_, where *m*_*x*_ is the magnetization component collinear with the current^14^. Because of inhomogeneity in film thickness and anisotropy in ultrathin CoFeB films^30^, with a medium *H*_*x*_ (145 Oe) applied, the moment may be tilted and induces a considerable *m*_*x*_ in the area with weak PMA, and thus the corresponding *H*_*SH*,*z*_ induces nucleation of the reversed domain, assisted by thermally activated processes^14^. In addition, we noticed that the domains always nucleate near the junction of Hall probe and the micro-stripe which is likely due to the higher demagnetization energy and reduced *H*_*k*_^*eff*^^31^. We believe the effect of width modulation on current density distribution also contribute to the nucleation at the crosspoints of the Hall probes and the micro-stripe. Differing from the case with small *H*_*x*_, we observed a distinct transverse DW expansion induced by the current pulses. However, the DW motion from the bottom to top edge is much faster than that in the opposite direction, indicating that the Oersted field cannot be ignored completely. Finally, with a medium *H*_*x*_ applied, much of the area in the Hall bar was reversed after 20 current pulses [Fig. 4(h)], which corresponds to a deterministic switching in the Fig. 1. These results also demonstrate that the required *H*_*x*_ does not need to be larger than H_DMI_ for deterministic current-induced switching, which may depend on the nucleation location of the reversed domain in the devices.

**Figure 5:**
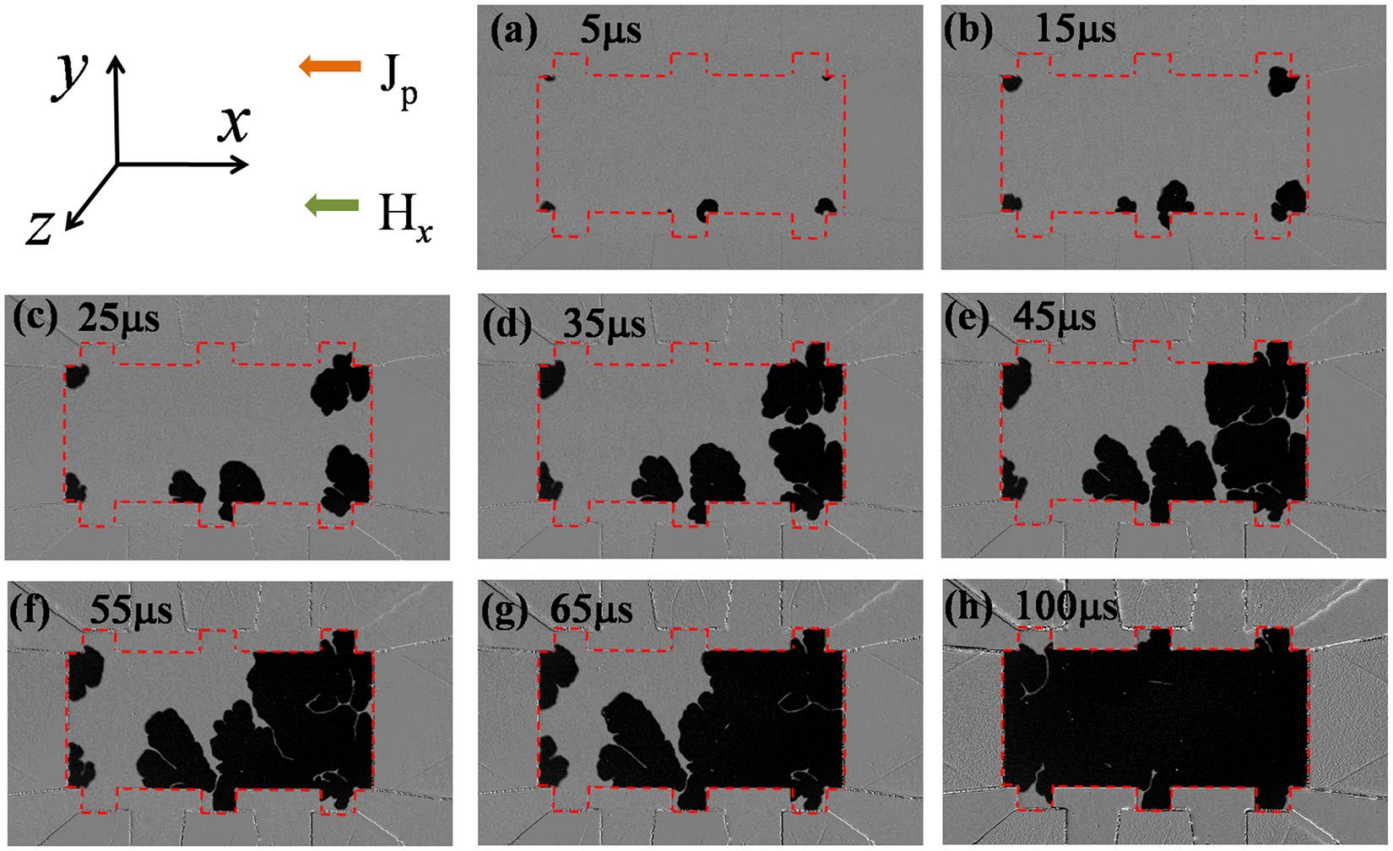
Same as Fig. 3 but in the presence of a large *H*_*x*_ of −1000 Oe. The device was pre-saturated with a downward magnetization and negative current flowing leftward. The magnitude of current density was reduced to 5.5 MA/cm^2^ to observe the current-induced reversal process.

With *H*_*x*_ = −1kOe and *J*_*p*_ = 10 MA/cm^2^, the current-induced magnetization reversal was completed in a single pulse because of the large *H*_*SH*,*z*_ and DW motion velocity. For further insight into this reversal process with large *H*_*x*_, we reduced *J*_*p*_ to 5.5 MA/cm^2^ and observed the reversal process using MOKE imaging (Fig. 5). Similar to a medium *H*_*x*_, domains had nucleated on both edges of the stripe near the voltage electrodes. Although the expansion of the reversed domain exhibited irregularity due to inhomogeneity in the films, we did not see obvious directionality in the DW motion in the transverse direction; this differs from that with small and medium *H*_*x*_ (Figs 3 and 4). We observed an almost isotropic DW propagation induced by the current pulses and ultimately a complete reversal of the entire magnetic area within 20 current pulses, as shown in Fig. 5(h).

## Discussion

**Figure 6:**
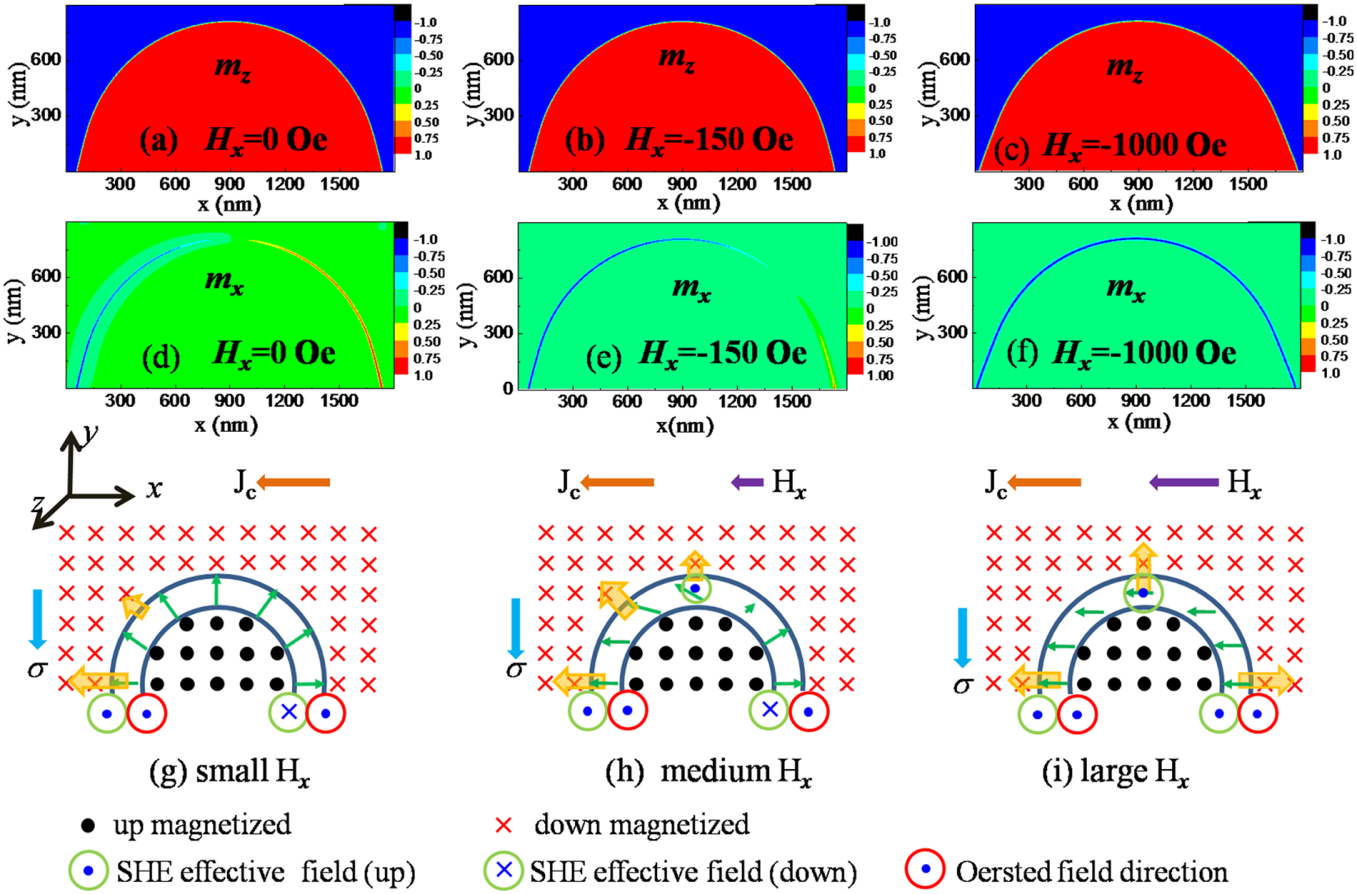
Micromagnetic simulation of the DW structure in Ta/CoFeB/MgO structures with DMI effect. (**a**–**f**) *m*_*z*_ and *m*_*x*_ distributions obtained from the simulations for different *H*_*x*_. (**g**–**i**) schematics of the magnetic moment orientation near the DW and corresponding SHE effective field and Oersted field induced by the current.

To understand the difference in the current-induced DW propagation process under the various *H*_*x*_, we performed a micromagnetic simulation on the DW structure of the device. We chose a 900 × 1800 nm rectangular area, in which a semi-circular reversed domain formed at the bottom edge with magnetization pointing up (+z direction) and other area with magnetization pointing down (−z direction). Figure 6(a–f) shows the *z*-component (*m*_*z*_) and *x*-component (*m*_*x*_) of the magnetic moment obtained from simulations for various *H*_*x*_. Because *H*_*x*_ is much smaller than the PMA of the films, it did not significantly affect *m*_*x*_ and *m*_*z*_ in the domains, but rather the orientation of the moment in the DW. Without *H*_*x*_ applied, the distribution of *m*_*x*_ [Fig. 6(d)] and *m*_*y*_ (not shown) in the DW confirmed the Néel wall profile resulting from the DMI. It should be noted that the effect of field-like torque on the spin configuration in DWs was neglected in the simulation because its amplitude (about 22 Oe for the given current density *J*_*p*_ = 10 MA/cm^2^) is much lower than the *H*_*DMI*_ and the in-plane field (*H*_*x*_).

Following an analysis of the vertical component of the effective SOT field at the DW, the current-induced DW propagation can be explained. According to the previous discussions, the vertical component of the SHE equivalent field produced by a negative current (−x direction) can be expressed as *H*_*SH*,*z*_ = *H*_*SH*_*m*_*x*_. Therefore, *H*_*SH*,*z*_ experienced by the DW depends not only on the amplitude and direction of the current density *J*, but also on the orientation $\hat{m}$ of the magnetization within the DW. Without *H*_*x*_, the direction of *H*_*SH*,*z*_ on both sides of the half-circular DW are opposite because of the Néel wall profile, whereas the Oersted field has the same amplitude and direction. As a result, the total effective field is enhanced on the left-hand side of the DW and is canceled on the right-hand side, as illustrated in Fig. 6(g). The enhanced effective field can overcome the pinning field and induce a leftward DW motion, whereas on the right-hand side the DW is still pinned because of the small effective field. In addition, in the top portion of the circular DW, *H*_*SH*,*z*_ is nearly zero because *m*_*x*_ is nearly zero and therefore no transverse DW propagation is observed. This is consistent with the experimental observation (Fig. 3)

Figure 6(b,e) and (h) shows the *m*_*z*_ and *m*_*x*_ values, respectively, obtained from the simulation and a schematic of the corresponding DW magnetization for the medium external field *H*_*x*_ = −150 Oe, which is about half of the measured *H*_DMI_ corresponding to the experimental conditions in Fig. 4. In this case, the external field is thus not sufficient to overcome the DMI field but does change the orientation of the moment in the DW. We note that the magnetic moments on both sides of the DW retain their original orientation and therefore a leftward DW expansion similar to that without *H*_*x*_ is observed. In the top part of the DW, the non-zero *m*_*x*_ may induce a considerable *H*_*SH*,*z*_ and a corresponding transverse DW motion along the *y*-direction, confirmed by the experimental observations in Fig. 4. Because both longitudinal and transverse DW motion occurs, the reversed domain can expand in both *−x* and *y* direction. However, because of the small net effective field at the right-hand side of the DW, no DW motion rightwards is observed and a small magnetic area at the right-hand side of the stripe was not reversed even after 20 current pulses (100 *μs*), as illustrated in the Fig. 4(h).

With the *m*_*z*_ and *m*_*x*_ values obtained from simulations and from the corresponding schematic for a large external field *H*_*x*_ = −1000 Oe [Fig. 6(c,f and i)], the applied field fully overcomes *H*_DMI_ and completely aligns the moment in the DW along the −x direction. Therefore, *H*_*SH*,*z*_ is always pointing up along the DW, which induces an isotropic DW expansion in all lateral directions and ultimately results in complete magnetization reversal. The theoretical expectation is consistent with experimental results (Fig. 5).

In summary, we have studied magnetization reversal driven by SOT and the DMI in the Ta/CoFeB/MgO structure. The results suggest that for as-deposited Ta/CoFeB/MgO structure, the DMI effective field was found to be around 300 Oe, which stabilized the chiral Néel walls. In such a structure, SOT-induced magnetization reversals exhibit different behavior under various *H*_*x*_. With a small *H*_*x*_ applied, the Oersted field governed the nucleation at an edge of the stripe, and the current-induced DW motion is unidirectional because of the chiral Néel DW. For medium *H*_*x*_ (<H_DMI_), due to the increase of the spin Hall effective field and the change of DW configuration, the magnetization reversal is fulfilled by the nucleation at both edges of the stripe and current-induced asymmetric DW motion. In applying larger *H*_*x*_ (>H_DMI_), that overcame the chiral Néel wall and aligned substantially the moment in the DW along the field direction, the spin Hall field expanded the reversed domain in all lateral directions and induced a complete magnetization switching. The results also suggest that the required *H*_*x*_ for SOT-induced complete switching is not necessarily larger than H_DMI_ because of the transverse DW motion with a medium *H*_*x*_ applied.

## Methods

### Sample preparation

The film stack with the structure of Ta (3 nm)/Co_20_Fe_60_B_20_ (1.3 nm)/MgO (1 nm)/Ta (1 nm) layers was deposited at room temperature on thermally oxidized Si substrates by using a magnetron sputtering system with a base pressure below 1.0 × 10^−7^ Torr. Ar (5 mTorr) gas was used during the sputtering process. The Ta and CoFeB layers were grown by direct-current sputtering and the MgO layer was grown by radio-frequency sputtering using a ceramic MgO target. The film stack was subsequently patterned into eight-terminal Hall bar devices of differing dimensions by standard photolithography and ion milling techniques. Finally, Al(300 nm)/TiWN(10 nm) electrodes were formed at the ends of the channel and Hall probes.

### Micromagnetic simulation

The magnetic moment orientation in the domain and DW was calculated by solving the Landau-Lifshitz-Gilbert equation, given as

1 $$\frac{d\hat{m}}{dt}=-\gamma \hat{m}\times {H}_{eff}+\alpha \hat{m}\times \frac{d\hat{m}}{dt}$$

where $\hat{m}$ is the unit vector along the magnetization, α (=0.3) the damping constant, $\hat{z}$ the unit vector along the thickness direction, *H*_*eff*_ the effective field including the exchange, magnetostatic, anisotropy and DMI. The contribution of DMI to the total *H*_*eff*_ can be expressed as^32^:

2 $${\overrightarrow{H}}_{DMI}=\frac{2D}{{\mu }_{0}{M}_{{\rm{s}}}}[(\overrightarrow{\nabla }\cdot \overrightarrow{m})\hat{z}-\overrightarrow{\nabla }{m}_{z}]$$

In the calculation, the cell size is 3 nm × 3 nm × 0.8 nm with a total of 600 × 300 × 1 cells, and an exchange constant of 16 pJ/m was used. The values of other parameters for the simulation were obtained from experimental results: specifically, *Ms* = 1200 emu/cc, *H*_*k*_ = 22 kOe, DMI = 0.22 mJ/m^2^, and *t*_CoFeB_ = 0.8 nm after subtracting the dead layer thickness at Ta/CoFeB interface^33^.

### Data Availability

The datasets generated during the current study are available from the corresponding author on reasonable request.
